# Nomogram to Predict Left Atrial Thrombus or Spontaneous Echo Contrast in Patients With Non-valvular Atrial Fibrillation

**DOI:** 10.3389/fcvm.2021.737551

**Published:** 2021-10-14

**Authors:** Zhitong Li, Quanbo Liu, Fei Liu, Tesfaldet H. Hidru, Yuqi Tang, Tao Cong, Lianjun Gao, Xiaolei Yang, Yunlong Xia

**Affiliations:** ^1^Department of Cardiology, First Affiliated Hospital of Dalian Medical University, Dalian, China; ^2^Department of Respiratory Medicine, Shengjing Hospital of China Medical University, Shenyang, China

**Keywords:** left atrial thrombus, spontaneous echo contrast, atrial fibrillation, nomogram, risk score

## Abstract

**Background:** The predictive power of the CHADS_2_ and CHA_2_DS_2_-VASc scores for the presence of Left atrial thrombus (LAT)/ spontaneous echo contrast (SEC) in non-valvular atrial fibrillation (NVAF) is modest. The aim of this analysis is to define clinical and ultrasonic variables associated with LAT/SEC and to propose nomograms for individual risk prediction.

**Methods:** Data on 1,813 consecutive NVAF patients who underwent transesophageal echocardiography (TEE) from January 2016 to January 2021 were collected. The univariate and multivariate logistic regression analyses were used to construct a nomogram. We examined the predictive ability of the risk scores by calculating the area under the curve (AUC). Moreover, the performance of the nomogram was assessed with respect to calibration, discrimination, and clinical usefulness.

**Results:** LAT/SEC was found in 260 (21.0%) and 124 (21.6%) patients in the training and validation cohorts, respectively. On multivariate analysis, independent factors for LAT/SEC were Age, left atrial diameter (LAD), left ventricular ejection fraction (LVEF), hypertension (HTN), previous stroke or transient ischemic attack, Non-paroxysmal AF and a nomogram was built based on these variables. The calibration curve for the probability of LAT/SEC showed good prediction agreement with actual observation. The nomogram achieved good concordance indexes of 0.836 and 0.794 in predicting LAT/SEC in the training and validation cohorts, respectively. Decision curve analysis demonstrated that the nomogram would be clinically useful.

**Conclusions:** In this study, a nomogram was constructed that incorporated six characteristics of NVAF patients. The nomogram may be of great value for the prediction of LAT/SEC in NVAF patients.

## Introduction

Atrial fibrillation (AF) is the most common cardiac arrhythmia and is associated with a 5-fold risk for stroke (1). Recent evidence stated left atrial thrombus (LAT) and left atrial spontaneous echo contrast (LASEC) as risk factors of cardiogenic embolism in atrial fibrillation patients (2). Although transesophageal echocardiography (TEE) is still considered the gold standard to exclude LA/LAA thrombus, TEE requires special skills for proper performance and interpretation. Additionally, it is a relatively invasive test, usually performed with the patient under conscious sedation. Therefore, a potentially non-invasive and efficacious method allowing identification of LAT/SEC with reliability and accuracy comparable to TEE would be of significant clinical value.

The current guidelines for anticoagulant therapy for stroke prevention in NVAF depend on CHADS_2_ and CHA_2_DS_2_-VASc scores (3). However, the predictive power of the CHADS_2_ and CHA_2_DS_2_-VASc scores for the presence of LAT in NVAF is not satisfactory (c-statistics 0.55~0.70) (4, 5). Thrombus can be found even in some patients with low CHA2DS2-VASc scores (6). As such, it is of scientific interest to establish a stronger predictive model that incorporates factors associated with LAT/SEC based on clinical and ultrasonic data.

A powerful model that estimates LAT/SEC presence can assist cardiologists to identify high-risk patients and lead to a rational therapeutic choice. As a result, many efforts on the peri-procedural estimation of LAT/SEC have been made previously (5, 7). However, there is still no accurate model to predict LAT/SEC. Owing to this lack of a specific and practical predictive method, the development of a predictive model that incorporates factors associated with LAT/SEC based on peri-procedural clinicopathologic data becomes desirable.

In this study, we applied nomogram analysis, which can provide individualized, evidence-based, and highly accurate risk estimation. To our knowledge, we have established the first nomogram for peri-procedural LAT/SEC risk estimation in NVAF. The objectives of this study include to (1) investigate the predictive power of the CHADS_2_ and CHA_2_DS_2_-VASc scores for the presence of LAT/SEC, (2) identify the clinical predictors of LAT/SEC, and (3) establish nomogram for LAT/SEC risk estimation in NVAF.

## Methods

### Study Participants

We retrospectively enrolled 1,899 consecutive patients with non-valvular AF who underwent a TEE from January 2016 to January 2021 in the First Affiliated Hospital of Dalian Medical University (FAHDMU). Patients who were referred for catheter ablation or direct current cardioversion underwent transesophageal echocardiography (TEE) were eligible for this study. Patients with organic valvular heart diseases, rheumatic heart disease, prosthetic valve placement, malignant tumor were excluded. Likewise, individuals with missing/incomplete echocardiography or laboratory data were excluded from the analysis. Finally, 1,813 eligible patients were randomly assigned into the training cohort (*n* = 1,239) and validation cohort (*n* = 574). The study was approved by the first affiliated hospital of the Dalian medical University institutional review board, and the requirement for informed consent was waived. The research was conducted in accordance with the Helsinki declaration guidelines and all procedures listed here were carried out in compliance with the approved guidelines.

### Definition of the Explanatory Variables

Data on demographics, medical history, and laboratory data, and medications were collected from the electronic medical record of FAHDMU. All anticoagulants were administered at least 5–7 days until the TEE day. Diabetes mellitus was defined as a fasting glucose level ≥126 mg/dL (or non-fasting glucose ≥200 mg/dL), a physician diagnosis of diabetes, or use of diabetes medications (8). Congestive heart failure was defined as clinical heart failure (stage C or D) according to the ACC/AHA guidelines (9). Prevalent coronary artery disease (CAD) was defined by a history of physician-diagnosed myocardial infarction, coronary artery bypass surgery, or coronary angioplasty. Hypertension (HTN) was defined as systolic blood pressure ≥ 140 mmHg or diastolic blood pressure ≥ 90 mmHg at two or more visits or a past medical history of hypertension (10). The definition and classification of AF were according to the published guideline (3). Non-paroxysmal AF was composed of persistent, long-standing persistent, and permanent AF.

### Assessment of CHADS2 and CHA2DS2-VASc Score and Risk Classification

CHADS_2_ score was determined by assigning 1 point each for the presence of congestive heart failure (CHF), hypertension, age ≥ 75 years, and diabetes and by assigning 2 points for the previous stroke/transient ischemic attack (TIA). The CHA_2_DS_2_-VASc score was determined by assigning 1 point each for the presence of CHF, hypertension, age 65–74 years, diabetes, and vascular disease (peripheral artery disease or myocardial infarction) and by assigning 2 points each for age ≥ 75 years and previous stroke/TIA (11, 12). Current guidelines recommend anticoagulation for all patients with documented atrial fibrillation and a CHA_2_DS_2_-VASc score of 2 or greater in men and 3 or greater in women (3). Therefore, we classified men with a CHA_2_DS_2_-VASc score of 0–1 or women with a CHA_2_DS_2_-VASc score of 0–2 as low risk.

### Ultrasound Evaluation

All patients routinely underwent transthoracic echocardiography and TEE before catheter ablation or direct current cardioversion. Transthoracic echocardiography was performed with a Vivid 7 ultrasound system (GE Healthcare, Waukesha, WI, USAGE Vingmed Ultrasound) and an M3S probe for the subjects in partial left decubitus. TEE was performed with an HP Sonos5500 color Doppler flow imager using a multi-planar transesophageal ultrasound probe frequency of 4–7 MHz and suitable gain adjustment. The probe was advanced to the mid-esophagus, 25–35 cm from the incisor teeth. A multi-axial scan was performed on the horizontal section of the left heart to display the LAA and then a 0–180° continuous scan was performed at different angles and depths to maximize visualization of the structure of the LAA and its internal echoes. Before the patients underwent the TEE examination, the procedure was explained in detail, and written informed consent was obtained from all patients. Thrombus was defined as a circumscribed, uniformly echo dense mass distinct from the underlying left atrial endocardium and pectinate muscles detected in more than 1 imaging plane. Spontaneous echocardiographic contrast (SEC) was defined as dynamic “smoke-like” echoes with a characteristic swirling motion that could not be eliminated despite optimized gain settings (2). All measurements were performed and interpreted by experienced physicians who were blind to the study.

### Statistical Analysis

Continuous data were expressed as mean (SD) and compared using an unpaired, 2-tailed *t*-test, or Mann–Whitney test. The categorical data were presented as count and percentage and analyzed by χ^2^ test or Fisher exact test. Prior to the data analysis, patients with NVAF were divided into the following two groups according to their AF status: patients with paroxysmal AF and patients with Non-paroxysmal AF.

The significance of each variable in the training cohort was assessed by univariate logistic regression analysis for investigating the independent risk factors of the presence of LAT/SEC. All variables associated with LAT/SEC at a significant level were candidates for stepwise multivariate analysis. Further, a nomogram was formulated based on the results of multivariate logistic regression analysis using the rms package of R, version 4.0 (http://www.r-project.org/). The nomogram is based on proportionally converting each regression coefficient in multivariate logistic regression to a 0–100-point scale. The effect of the variable with the highest β coefficient (absolute value) is assigned to 100 points. The points of the independent variables were added to derive total points, which were converted to predicted probabilities. The predictive performance of the nomogram was evaluated by concordance index (C-index) and calibration with 1,000 bootstrap samples to decrease the overfit bias. Decision curve analysis was conducted using the R library rmda package to determine the clinical usefulness of the nomogram by quantifying the net benefit at different threshold probabilities in the primary dataset.

Receiver operating characteristic curve analysis was used to investigate the optimal cutoff values that were determined by maximizing the Youden index (sensitivity + specificity – 1). The accuracy of the optimal cutoff value was assessed by the sensitivity, specificity, predictive values, and likelihood ratios. *P*<.05 was considered statistically significant. All analyses were performed using R software, version 4.0.

## Results

During the study period, 1,813 consecutive NVAF patients were collected. The patients were divided into the training (1,239, 68.3%) and validation cohorts (574, 31.7%). The baseline data were similar between the training and validation cohorts. The mean age of the participants was 62.6 ± 9.4 years. Of the total participants, 64.4% were males. LAT/SEC was found in 260 (21.0%) and 124 (21.6%) patients in the training and validation cohorts, respectively. The clinical and demographic characteristics of the patients are presented in Table 1.

**Table 1:** Baseline characteristics.

**Variable**	**Cohort**		***P*-value**
	**Training (*n* = 1,239)**	**Validation (*n* = 574)**	
Age, years	62.6 (9.5)	62.6 (9.3)	0.987
Male sex, *n* (%)	811 (65.5)	356 (62.0)	0.171
**Medical history**			
HTN, *n* (%)	767 (61.9)	361 (62.9)	0.725
T2DM, *n* (%)	297 (24.0)	140 (24.4)	0.893
Previous stroke/TIA, *n* (%)	248 (20.0)	96 (16.7)	0.11
Vascular disease, *n* (%)	39 (3.1)	14 (2.4)	0.494
Non-paroxysmal AF, *n* (%)	445 (35.9)	206 (35.9)	1
CAD, *n* (%)	402 (32.4)	174 (30.3)	0.394
CHADS_2_ Score	1.39 (1.17)	1.34 (1.13)	0.415
CHA_2_DS_2_-VASc score	2.46 (1.45)	2.38 (1.43)	0.25
**laboratory data**			
eGFR, ml/(min 1.73 m^2^)	90.1 (18.8)	90.7 (19.0)	0.527
Uric acid, μmol/L	366.99 (87.77)	368.09 (91.70)	0.807
PT-INR	1.21 (0.50)	1.27 (0.58)	0.019
**Echocardiographic parameters**			
LAT/SEC, *n* (%)	260 (21.0)	124 (21.6)	0.812
LAD, mm	39.2 (4.5)	39.1 (4.6)	0.729
LVEDD, mm	47.9 (4.3)	47.9 (4.2)	0.81
LVEF, %	56.5 (4.9)	56.3 (5.2)	0.443
**Medication**			
Statin, *n* (%)	752 (60.7)	334 (58.2)	0.336
Amiodarone, *n* (%)	908 (73.3)	432 (75.3)	0.404
Antiplatelet, *n* (%)	378 (30.5)	159 (27.7)	0.245

*CAD, coronary artery disease; HTN, Hypertension; LAD, left atrium diameter; LAT, left atrial thrombus; LVEDD, left ventricular end diastolic diameter; LVEF, left ventricular ejection fraction; SEC, spontaneous echo contrast; T2DM, Type 2 diabetes mellitus; TIA, transient ischemic attack*.

The results of the univariate and multivariate logistic analysis are presented in Table 2. In the LAT/SEC group, patients had a higher prevalence of hypertension, prior stroke/TIA, non-paroxysmal AF, CAD, and larger left atrial diameter. Likewise, the LAT/SEC group had higher values of CHADS_2_ and CHA_2_DS_2_-VASc scores, but lower left ventricular ejection fraction and estimated glomerular filtration rate. However, the prevalence of vascular disease and diabetes mellitus was similar in the two groups. The multivariate analysis showed that risk factors such as age (OR = 1.04, 95% CI: 1.02–1.06, and *P* = 0.001), LAD (OR = 1.21, 95% CI: 1.16–1.26, and *P* = 0.001), LVEF (OR = 0.92, 95% CI: 0.89–0.94, and *P* = 0.001), previous stroke/transient ischemic attack (OR = 1.79, 95% CI: 1.23–2.60, and *P* = 0.002), hypertension (OR = 1.44, 95% CI: 1.02–2.06, and *P* = 0.041) and non-paroxysmal AF (OR = 2.76, 95% CI: 1.99–3.85, and *P* = 0.001) remained independently associated with LAT/SEC.

**Table 2:** Univariate and multivariate logistic regression analysis of LAT/SEC presence based on peri-procedural data in the training cohort.

**Variable**	**Univariate analysis**		**Multivariate analysis**	
	**OR (95%CI)**	***P*-value**	**OR (95%CI)**	***P-*value**
Age, years	1.03 (1.02–1.05)	<0.001	1.04 (1.02–1.06)	0.001
Male sex	0.95 (0.72–1.27)	0.748		
**Medical history**				
HTN	1.68 (1.25–2.27)	0.001	1.44 (1.02–2.06)	0.041
T2DM	1.13 (0.82–1.54)	0.445		
Previous stroke/TIA	1.94 (1.41–2.65)	<0.001	1.79 (1.23–2.60)	0.002
Vascular disease	1.93 (0.95–3.74)	0.058		
Non-paroxysmal AF	3.58 (2.7–4.77)	<0.001	2.76 (1.99–3.85)	0.001
CAD	1.34 (1.01–1.78)	0.042		
**laboratory data**				
eGFR, ml/(min 1.73 m^2^)	0.99 (0.98–0.99)	<0.001		
Uric acid, μmol/L	1.02 (1.01–1.04)	0.028		
PT-INR	1.5 (1.17–1.91)	0.001		
**Echocardiographic parameters**				
LAD, mm	1.27 (1.23–1.32)	<0.001	1.21 (1.16–1.26)	0.001
LVEDD, mm	1.09 (1.06–1.13)	<0.001		
LVEF, %	0.89 (0.86–0.91)	<0.001	0.92 (0.89–0.94)	0.001
**Medication**				
Statin	1.05 (0.79–1.39)	0.754		
Amiodarone	1.21 (0.88–1.67)	0.24		
Antiplatelet	1.02 (0.75–1.36)	0.918		

*CAD, coronary artery disease; HTN, Hypertension; LAD, left atrium diameter; LAT, left atrial thrombus; LVEDD, left ventricular end diastolic diameter; LVEF, left ventricular ejection fraction; SEC, spontaneous echo contrast; T2DM, Type 2 diabetes mellitus; TIA, transient ischemic attack*.

**Figure 1:**
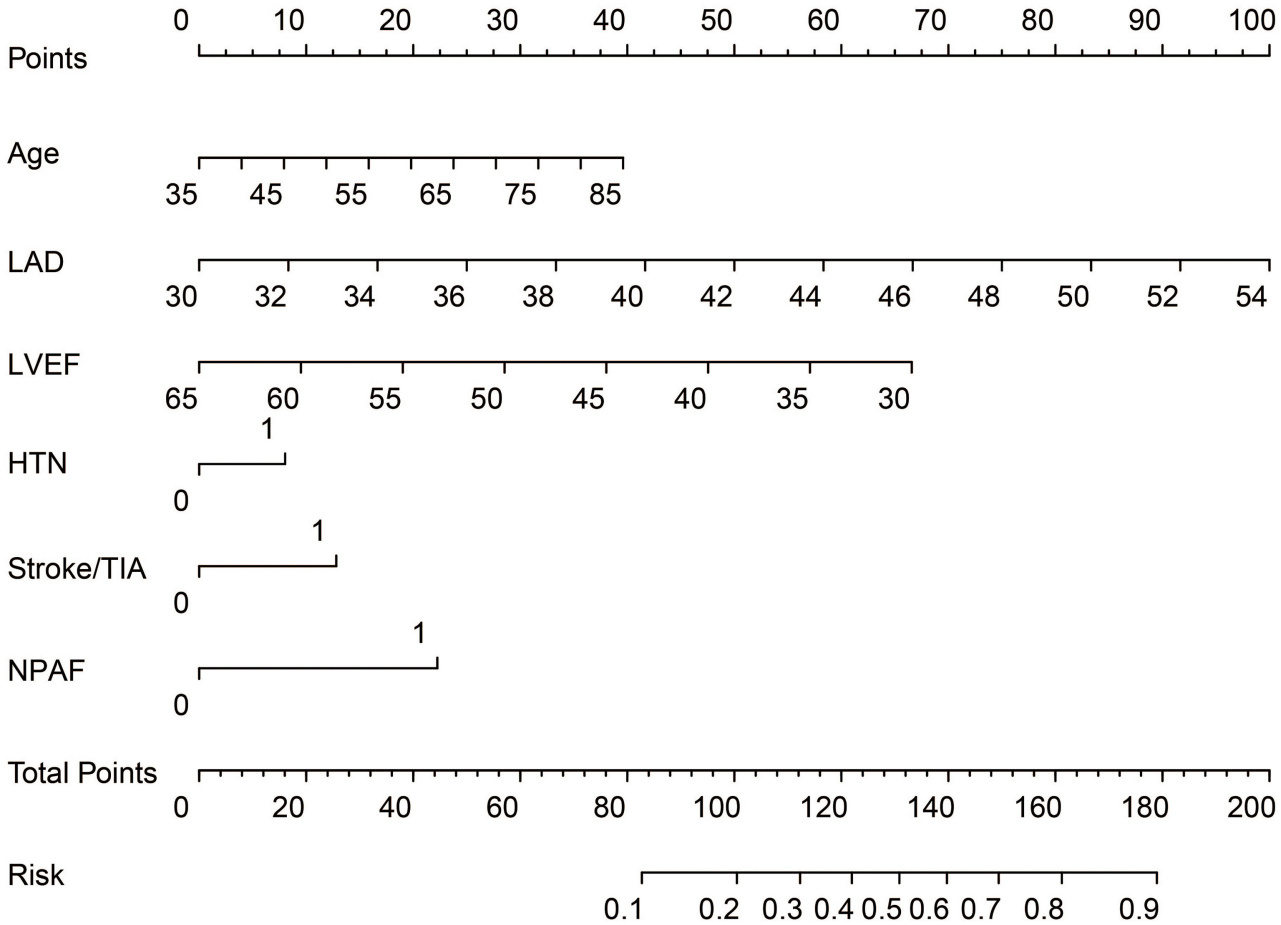
Nomogram for peri-procedural estimation of LAT/SEC risk in NVAF patients. HTN, Hypertension; LAD, left atrium diameter; LVEF, left ventricular ejection fraction; NPAF, Non-paroxysmal AF.

ROC curve analysis was used to investigate the predictive power of the CHADS_2_ and CHA_2_DS_2_-VASc scores concerning LAT/SEC. The results showed that the c-statistic of the CHADS_2_ and CHA_2_DS_2_-VASc scores were 0.608 and 0.606, respectively. Furthermore, we developed a LAT/SEC risk estimation nomogram based on the results of the multivariate logistic analysis (Figure 1). The bootstrap validation method was used to internally validate the resulting model.

**Figure 2:**
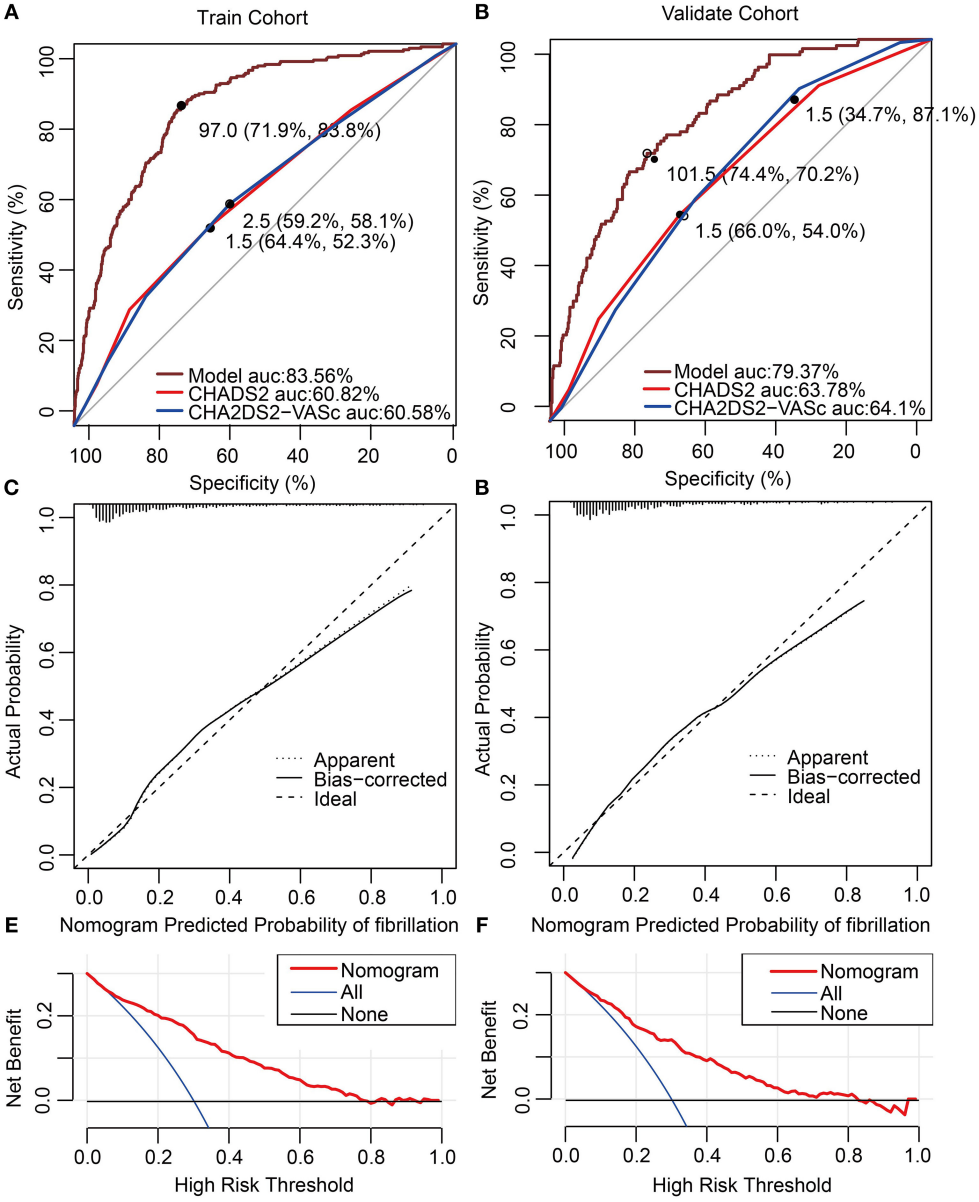
**(A,B)** Receiver operating characteristic curve for models in predicting LAT/SEC in the training cohort and validation cohort. **(C)** Calibration curves for the nomogram in the training cohort. The dotted line represents the entire cohort (*n* = 1,239), and the solid line is the result after bias-correction by bootstrapping (1,000 repetitions), indicating nomogram performance (boot mean absolute error = 0.027). **(D)** Calibration curves for the nomogram in the validation cohort. The dotted line represents the entire cohort (*n* = 574), and the solid line is the result after bias-correction by bootstrapping (1,000 repetitions), indicating nomogram performance (boot mean absolute error = 0.020). **(E,F)** Decision curve analysis for the nomogram in the training cohort and validation cohort. The decision curve of the nomogram is composed of an *X*-axis which represents continuum of potential thresholds for LAT/SEC risk and a *Y*-axis which represents the net benefit which is obtained by dividing the net true positives by the sample size. The “All” curve shows the net benefit if all patients subject to transesophageal echocardiography (TEE). The “None” line shows the net benefit if no patient subject to TEE. The “Nomogram” curve shows the net benefit if it is used to select patients for TEE. For example, if the personal threshold probability of a patient was 40%, the net benefit would be 0.1 when using the nomogram to decide whether to conduct TEE examination, which means that there are 10 net detected LAT/SEC per 100 patients.

The nomogram demonstrated a very good predictive power in estimating the risk of LAT/SEC, with an unadjusted C index of 0.836. Besides, calibration plots graphically showed good agreement on the presence of LAT/SEC between the risk estimation by the nomogram and TEE confirmation. In the validation cohort, the nomogram displayed a C index of 0.794 for the estimation of LAT/SEC risk. Also, our result indicates that the observed frequencies and the estimated probability of LAT/SEC presence showed a good calibration curve for the risk estimation (Figures 2A–D). The decision curve shows the clinical usefulness of the nomogram (Figures 2E,F). In this analysis, the final decision curve showed that for a threshold probability between 10 and 80%, the model had positive net benefit.

The optimal cutoff value of the total nomogram scores was determined to be 97.0. The sensitivity, specificity, positive predictive value, and negative predictive value were 83.8, 71.9, 44.2, and 94.4%, respectively in the training cohort, and 70.2, 74.4,43.1, and 90.1%, respectively in the validation cohort (Table 3).

**Table 3:** Accuracy of the prediction score of the nomogram for estimating the risk of LAT/SEC.

**Variable**	**Nomogram**		**CHADS_2_ score**		**CHA_2_D_2_VASc score**	
	**Training cohort**	**Validation cohort**	**Training cohort**	**Validation cohort**	**Training cohort**	**Validation cohort**
AUC	0.84	0.79	0.61	0.64	0.61	0.64
Cutoff score	97.0	101.5	1.5	1.5	2.5	1.5
Specificity, %	71.9	74.4	64.4	66.0	59.2	34.7
Sensitivity, %	83.8	70.2	52.3	54.0	58.1	87.1
NPV, %	94.4	90.1	83.6	83.9	84.2	90.7
PPV, %	44.2	43.1	28.0	30.5	27.5	26.9

*AUC, Area under ROC curve; NPV, Negative predictive value; PPV, Positive predictive value; ROC, receiver operating characteristic*.

## Discussion

The present study that established the first nomogram for LAT/SEC risk estimation in patients with NVAF found that a new model composed of age, LAD, LVEF, previous stroke or transient ischemic attack, HTN and non-paroxysmal AF had a better performance for the prediction of LAT/SEC compared to CHADS_2_ and CHA_2_DS_2_-VASc scores.

The prevalence of LAT/SEC for patients with AF varied in previous studies (2, 4, 13). In our study, the prevalence of LAT/SEC was 21.2% in NVAF population. In the present study, two variables that are not included in the CHA_2_DS_2_-VASc score were found to be the top predictors of LAT/SEC. Two predictors include LAD and non-paroxysmal AF. Therefore, it may be reasonable to consider NVAF patients with the enlarged left atrium and non-paroxysmal AF as candidates for more intensive medical follow-up. The association between these factors and LAT/SEC has also been reported in previous studies (7, 14–16). For instance, left atrial enlargement has been shown to associate with LAT/SEC, which is a surrogate marker of stroke risk (14, 17). Earlier evidence also reported that the possibility of thrombus formation increases with an enlarged LA cavity (14, 18). Although many mechanisms can explain the association between left atrial enlargement and thrombus formation, the mechanism that involves changes in left atrial hemodynamics, such as the existence of turbulences, reduced flow velocity, increased blood stasis and endothelial injury could be speculated as plausible mechanisms (19).

As earlier mentioned, non-paroxysmal AF remained a significant predictor for LAT/SEC in our study. Although the current ESC guidelines do not list AF type or AF burden among factors affecting the probability of LAA thrombus formation, few studies have shown that persistent or permanent AF carries a higher risk of stroke than paroxysmal AF (20). Relative to paroxysmal AF, non-paroxysmal AF shows greater structural remodeling and endocardial fibroelastosis of the atria and appendage, both of which are likely to contribute to thrombus formation (21, 22).

In our study, there were still 103 (14.9%) patients with LAT/SEC among the low-risk group (classified based on CHA_2_DS_2_-VASc score). In addition, the c-statistics of the CHADS_2_ and CHA_2_DS_2_-VASc scores were 0.608 and 0.606, respectively, suggesting CHADS_2_ and CHA_2_DS_2_-VASc scores had relatively weaker predictive performance in discriminating LAT/SEC compared to the new model. There could be two reasons that contribute to the observed phenomenon. Firstly, the models share the same risk factor with atherosclerosis. Consequently, CHADS_2_ and CHA_2_DS_2_-VASc scores may predict stroke through the mechanism of atherosclerosis but not *via* mechanisms that involve cardiogenic embolism. Secondly, these risk scores do not incorporate other risk factors that are highly linked to thrombo-embolic risks, such as echocardiographic components, biochemical concentrations, and coagulation parameters that are known for predisposing stasis of blood within the left atrium and appendage.

In this study, our multivariate analysis revealed several predictors of LAT/SEC. By combining these predictors of LAT/SEC, we constructed a nomogram model. Interestingly, the newly constructed model demonstrated a strong discriminatory performance to identify patients with increased risk of LAT/SEC. The prognostic relevance of such a model of clinical risk factors has not been prospectively studied in the past. According to our results, the discriminatory performance of the new composition score was even stronger than CHADS_2_ and CHA_2_DS_2_-VASc scores. For clinical use of the model, we recommend 97.0 as the cutoff value, and patients with a score of 97.0 or more should be considered as a high-risk group for LAT/SEC. Based on these predictions from the nomogram, the new model might serve as a substitute of TEE for NVAF patients who cannot tolerate TEE and provide references about whether to stop anticoagulants after procedural in the follow-up.

Our study has some limitations. First, this analysis was based on data from a single institution, thus it is necessary to validate the results from other centers. Second, this analysis is a retrospective study, some specific markers which might be associated with LAT/SEC such as left atrial appendage morphology, markers of endothelial dysfunction, and inflammation were not included in the nomogram. Moreover, the current study lacks data on left atrial volume, a more accurate marker to assess left atrial size. Third, the nomogram achieved a good predictive accuracy, with a cutoff point of 97.0, however, it demonstrated a significant proportion of false-positive and false-negative rates in the training (28.1 and 16.2%, respectively) and validation cohort (25.6 and 29.8%, respectively), which indicates replication of such study is of crucial importance in the future study to confirm the power of the utilized model in clinical decision making. Finally, the present study included only NVAF patients who underwent TEE before ablation or cardioversion intervention, therefore our results may be limited to NVAF patients who are candidates for ablation or cardioversion interventions.

## Conclusions

Our study showed that Age, LAD, LVEF, HTN, previous stroke or transient ischemic attack, and Non-paroxysmal AF were the risk factors of LAT/SEC from AF patients. By combining these risk factors of LAT/SEC, a nomogram was constructed. The model provides an optimal peri-procedural estimation of LAT/SEC risk in patients with non-valvular atrial fibrillation.

## Data Availability Statement

The raw data supporting the conclusions of this article will be made available by the authors, without undue reservation.

## Ethics Statement

The studies involving human participants were reviewed and approved by the First Affiliated Hospital of the Dalian Medical University Institutional Review Board. Written informed consent for participation was not required for this study in accordance with the national legislation and the institutional requirements.

## Author Contributions

XY and YX designed this study. ZL, QL, and YT were in charge of data analysis and data collection. ZL drafted the article. FL and TH did the critical revision of article. TC and LG conducted the data collection. All authors have read and approved the final manuscript.

## Conflict of Interest

The authors declare that the research was conducted in the absence of any commercial or financial relationships that could be construed as a potential conflict of interest.

## Publisher's Note

All claims expressed in this article are solely those of the authors and do not necessarily represent those of their affiliated organizations, or those of the publisher, the editors and the reviewers. Any product that may be evaluated in this article, or claim that may be made by its manufacturer, is not guaranteed or endorsed by the publisher.
